# Finite element study of sagittal fracture location on thoracolumbar fracture treatment

**DOI:** 10.3389/fbioe.2023.1229218

**Published:** 2023-08-07

**Authors:** Xilong Cui, Junjun Zhu, Wanmei Yang, Yuxiang Sun, Xiuling Huang, Xiumei Wang, Haiyang Yu, Chengmin Liang, Zikai Hua

**Affiliations:** ^1^ School of Mechatronics Engineering and Automation, Shanghai University, Shanghai, China; ^2^ Department of Orthopedics, Fuyang People’s Hospital, Fuyang, Anhui, China; ^3^ Spinal Deformity Clinical and Research Center of Anhui Province, Fuyang, Anhui, China

**Keywords:** thoracolumbar fracture, sagittal location, instrument, biomechanics, surgical strategy

## Abstract

**Background:** Posterior internal fixation is the main method used for the treatment of thoracolumbar fractures. Fractures often occur in the upper 1/3 of the vertebral body. However, they can also occur in the middle or lower 1/3 of the vertebral body. At present, there is no report discussing the potential effects of sagittal location on instrument biomechanics or surgical strategy. The object of this study was to investigate the effect of the sagittal location of the fracture region of the vertebral body on the biomechanics of the internal fixation system and surgical strategy.

**Methods:** A finite element model of the T11-L3 thoracolumbar segment was established based on a healthy person’s CT scan. Different sagittal fracture location finite element models were created by resection of the upper 1/3, middle 1/3, and lower 1/3 of the L1 vertebral body. Three surgical strategies were utilized in this study, namely, proximal 1 level and distal 1 level (P1-D1), proximal 2 level and distal 1 level (P2-D1), and proximal 1 level and distal 2 levels (P1-D2). Nine fixation finite element models were created by combining fracture location and fixation strategies. Range of motion, von Mises stress, and stress distribution were analyzed to evaluate the effects on the instrument biomechanics and the selection of surgical strategy.

**Results:** In all three different fixation strategies, the maximum von Mises stress location on the screw did not change with the sagittal location of the fracture site; nevertheless, the maximum von Mises stress differed. The maximum rod stress was located at the fracture site, with its value and location changed slightly. In the same fixation strategy, a limited effect of sagittal location on the range of motion was observed. P2D1 resulted in a shorter range of motion and lower screw stress for all sagittal locations of the fracture compared with the other strategies; however, rod stress was similar between strategies.

**Conclusion:** The sagittal location of a fracture may affect the intensity and distribution of stress on the fixation system but does not influence the selection of surgical strategy.

## Introduction

According to an epidemiological study, the incidence of spinal fractures is approximately 32.8/100 000 (Liu et al., 2018). Most fractures occurs in the thoracic spine, followed by the lumbar- and cervical spine, accounting for 41.6%, 33.7%, and 24.6% respectively (den Ouden et al., 2019). More than 14.3% of cases are burst fractures (Denis, 1983). The thoracolumbar spine is the transition region from the fixed thoracic spine to the flexible lumbar. Therefore, the thoracolumbar segment is the region where fractures occur most frequently (Holmes et al., 2001). Fracture fragments can migrate the spinal canal and potentially cause spinal cord injury. The main focus in the treatment of thoracolumbar fractures is to restore spinal stability. Burst fractures involve the anterior and middle columns, which are considered unstable (Petersilge and Emery, 1996). Internal fixation is the most important surgical option for the treatment of unstable thoracolumbar fractures. Internal fixation could be divided into anterior, posterior, and combined anterior and posterior operations. Posterior transpedicular internal fixation is the most commonly used method. Short-segment, limited long-segment, and long-segment fixation has been previously reported in the literature (El et al., 2020; Girardo et al., 2021; Liang et al., 2020). These techniques have resulted in good clinical outcomes; however, instrument loosening and breakage can occur (Mu et al., 2022).

Finite element (FE) analysis offers the advantages of good repeatability and cost-effectiveness. Thus, it has been widely used to understand and optimise the different fracture fixations, mechanical testing, and spine fracture biomechanical research (Naoum et al., 2021; Guo et al., 2021; Wong et al., 2021). Numerous studies have compared the maximum stress and distribution of different fixation methods. However, not all internal instruments have the same fracture location as predicted in clinical practice.

Studies revealed that the location and size of upper endplate injury in the coronal plane affect internal fixation and vertebral body stress. Wang and Hu found that in cases with 4/5 endplate fractures, internal fixation should not be removed after surgery (Wang and Hu., 2020). In patients with spinal tumors, the location and size could also affect spinal biomechanics (Galbusera et al., 2018). However, whether the sagittal distribution of the fractures affects the stress of internal fixation or surgical strategy has not been reported in the literature. Clinically, some researches have observed this sagittal distribution. According to the AO classification, type A3.2 is divided into upper burst fractures, lower burst fractures, and lateral burst fractures (Rosenthal et al., 2018). In the Denis classification, compression fractures are divided into Types A, B, C, and D. The fractured region of Type B, C, and D primarily located in the upper, middle, and lower regions, accounting for 62.4%, 6.09%, and 15.2%, respectively. In burst fractures, upper fractures account for 49.2%, while lower fractures account for 6.8%. Some researchers have also classified the mechanical mechanisms behind these distribution patterns. (Guo and Li., 2019).Therefore, we hypothesized that the sagittal distribution of fractures may affect the level and position of maximum mechanical stress on the internal fixation suggesting that the fracture level must be taken into account when developing a treatment algorithm.

In this study, we resected the upper 1/3, middle 1/3, and lower 1/3 of the vertebral body to simulate the sagittal distribution of different sagittal fractures, A3 burst fractures according to Vaccaro et al. (2013) Three internal fixation strategies were used, namely, one proximal and distal segment (P1D1), two proximal and one distal segment (P2D1), and one proximal and two distal segments (P1D2). The objectives of this study were to 1) investigate the effects of fracture sagittal distribution on internal fixation biomechanics, and 2) determine its potential influence on the selection of internal fixation strategy.

## Materials and methods

The volunteer selection criteria: 18–30 years old, no history of spinal tumors, lower back pain, or spinal surgery, BMI 18.5–23.9 kg/m^2^. A 25-year-old male with a BMI of 20.2 kg/m^2^ was involved in the study with written informed consent. The study was approved by the Ethics Committee of Fu Yang Hospital (No, 2020-11, Anhui, China).

Computed tomography images of T10-L4 were obtained using a Brilliance 256 CT scanner (Philips Brilliance iCT256, Eindhoven, Netherlands). The slice thickness was 0.5 mm and the in-plane resolution was 512 × 512. A three-dimensional model of T11-L3 was established with Mimics 21.0 (Materialise, Leuven, Belgium). The cortical bone and cancellous bone were based on geometry using the “Threshold” and “Regional Growth” tools. The mesh structure was prepared using the preprocessing software Geomagic Studio 12.0 (Geomagic, Cary, NC, United States). Notably, the thickness of the cortical bone and endplate was 1 mm and 0.5 mm, respectively (Wong et al., 2003).

UGNX12.0 (Dassault Systèmes, S.A, Paris, France) was used to construct the intervertebral disc. The nucleus pulposus and annular fibers were constructed separately. Additionally, the volume ratio of the annulus fibrosus to the nucleus pulposus was set to 6:4 (Wang et al., 2013).

A baseline three-dimensional FE model of a healthy T11-L3 was created first. The following three interfaces were modelled as bonded: the vertebral body-endplate, endplate-nucleus pulposus, and nucleus pulposu-annulus fibrosus. Moreover, frictionless contact was used to simulate the sliding contact between articular cartilages. The model was assumed to be homogeneous, isotropic, and linearly elastic. The material properties used are presented in Table 1 (Park et al., 2013; Wang et al., 2018). The ligaments (i.e., the anterior longitudinal ligament, posterior longitudinal ligament, ligamentum flavum, capsular ligament, and interspinous ligament) were constructed as nonlinear spring elements in ANSYS Workbench (Ansys, Pittsburgh, PA, United States), and the material properties are shown in Table 2(Rohlmann et al., 2009). After mesh convergence analysis, a total of 68,619 elements and 1,240,899 nodes were included.

**TABLE 1:** Material properties assumed for different components of the finite element (FE) model.

Spinal site	Young’s modulus (MPa)	Poisson’s ratio
Vertebra		
Cortical	12,000	0.3
Cancellous bone	100	0.2
Endplate	23.8	0.4
Cartilage	11	0.4
Intervertebral disc		
Nucleus pulposus	1	0.49
Annulus fibrosis	4.2	0.4
Pedicle screws and rods	110,000	0.3

**TABLE 2:** Material properties assumed for different components of ligaments.

Ligament	Rigidity	Strain ε (%)	Rigidity	Strain ε (%)	Rigidity	Strain ε (%)
Anterior	347	0–12.2	787	12.2–20.3	1,864	20.3
Posterior	29.5	0–11.1	61.7	11.1–23	236	23
Capsular	36	0–25	159	25–30	384	30
Intertransverse	0.3	0–18.2	1.8	18.2–23.3	10.7	23.3
Flavum	7.7	0–5.9	9.6	5.9–4.9	58.2	49
Supraspinal	2.5	0–20	5.3	20–25	34	25
Interspinal	1.4	0–13.9	1.5	13.9–20	14.7	20

### Model creation for different sagittal fracture distribution

**FIGURE 1:**
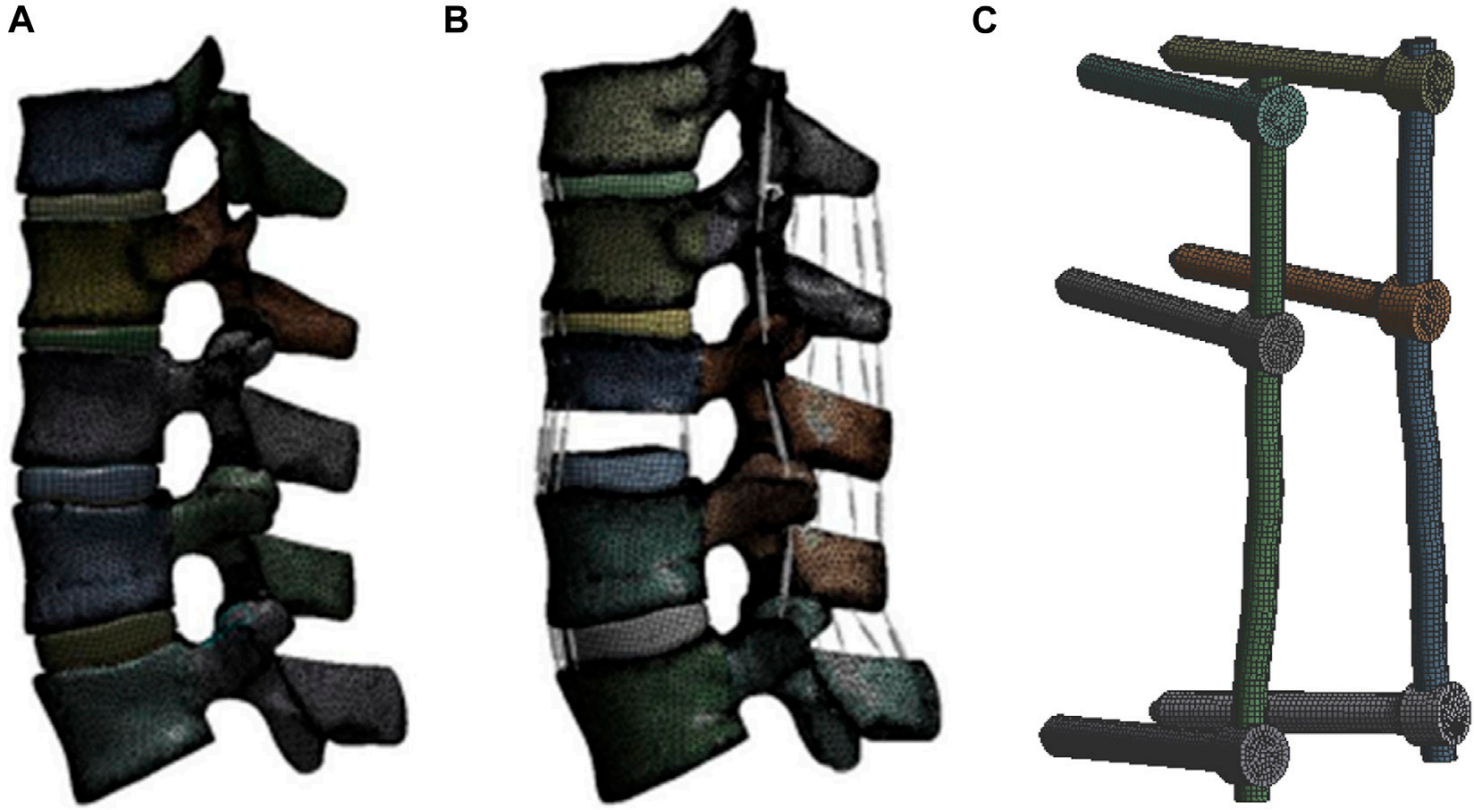
Finite element models with L3 fixed in all degree of freedom **(A)**, **(B)** 7.5 N*M moment in extension, flexion and lateral bending, **(C)** 5.5 N*M moment in axial rotation was assumed with 400 N compressive load: intact model; lower 1/3 L1 fracture model; and fixation instrument model.

The fractured vertebra model was created using SolidWorks (Simulia, United States). The upper, middle, and lower 1/3 of L1 were resected, and the posterior structure was maintained to establish an unstable type A3.2 thoracolumbar fracture, according to the AO spinal fracture classification (Vaccaro et al., 2013) (Figure 1).

### Creation of the pedicle and screw and rod models

Titanium alloy pedicle screws (6 mm × 50 mm for lumbar; 5.5 mm × 45 mm for thoracic vertebra) and rods (5.5 mm) were modeled using SolidWorks. The screws were inserted into the vertebra and connected with rods (Figure 1). Bonded contact was used between the screw and the vertebra, as well as between the screw and the rod. The mesh size was set to 1 mm for each screw and rod, and the unit included a total of 42,923 elements and 152,473 nodes.

### Models of different surgical strategies

**FIGURE 2:**
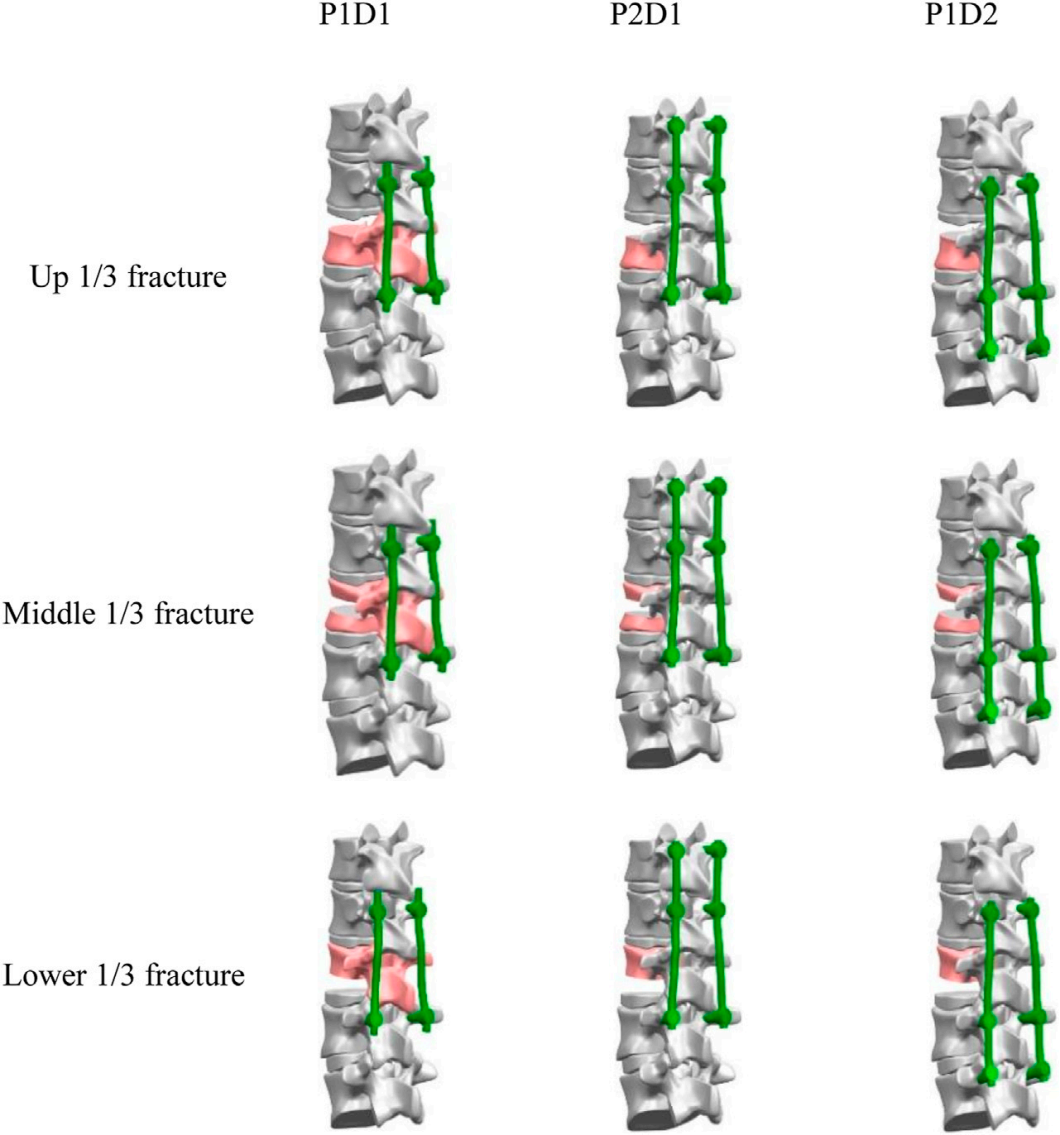
Models of different surgical strategies.

The models included upper 1/3 fracture, (U-P1D1, U-P2D1, U-P1D2), middle 1/3 fracture, (M-P1D1, M-P2D1, M-P1D2), and lower 1/3 fracture (L-P1D1, L-P2D1, L-P1D2) (Figure 2). Nine models were created in total.

### Load and boundary

The load and boundary conditions were based on research published by Basaran et al. (2019). The L3 vertebra body was fixed in all degree of freedom. A compressive load of 400 N was applied to the top surface of T11 was applied to all the models as follower loading. Movement in coronal, sagittal, and transverse planes were evaluated, including extension, lateral bending, and rotation motions. The extension, flexion and lateral flexion moments were assumed to be 7.5 N*M, while the axial rotation moment was assumed to be 5.5 N*M.

### Measurements and assessment indices

The range of motion (ROM) of T12-L2 was assessed in the nine FE models under six loading conditions. Data for the maximum von Mises stress and location were also collected and analyzed.

## Results

### Validation

**FIGURE 3:**
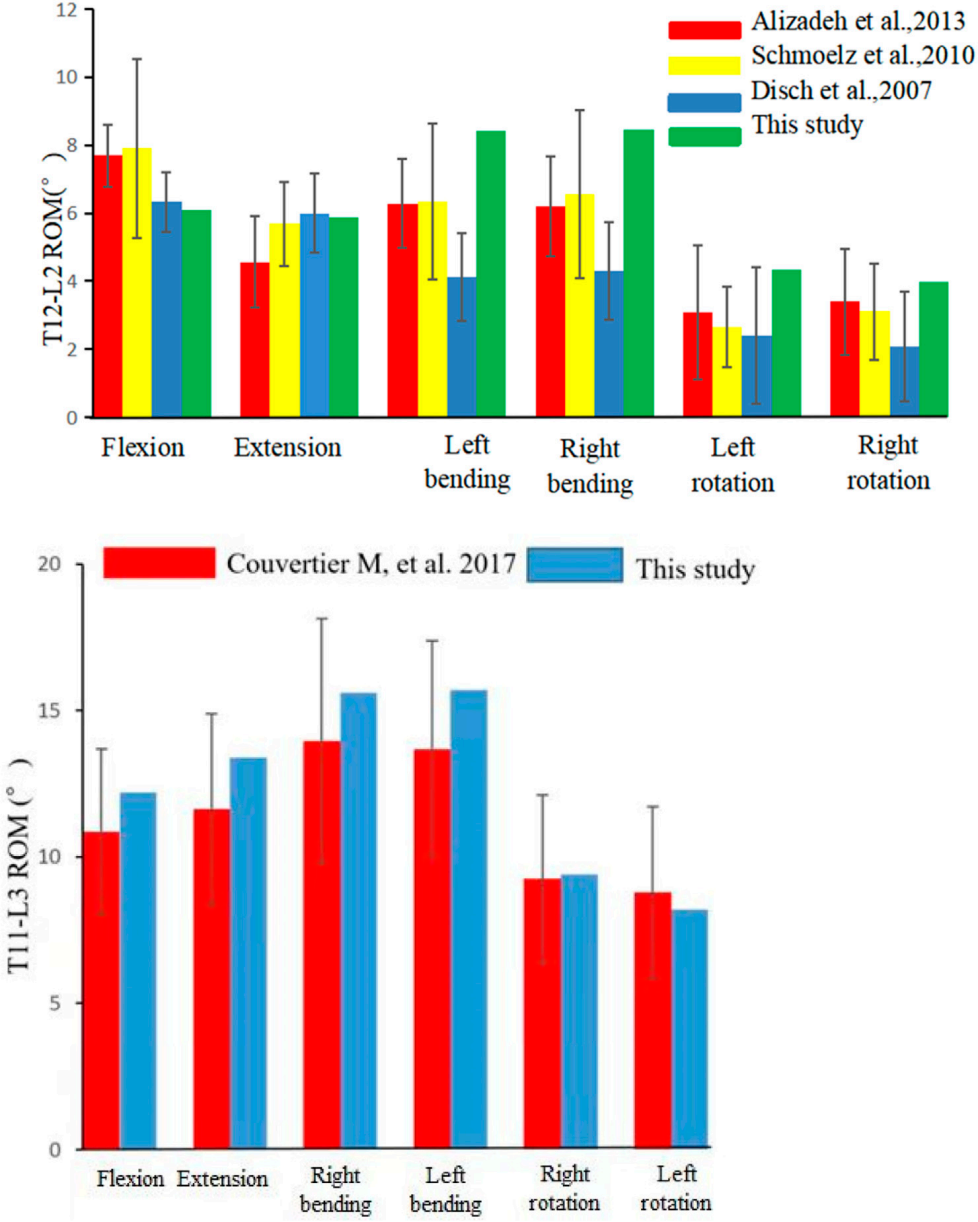
Comparison of the range of motion (ROM) of T12/L2 and T11-L3 in this study with those recorded in other studies.

Following the creation of the normal T12-L2 FE model, data on movement induced by 7.5 N*M in flexion, lateral bending and rotation were collected. The ROM values of the T12-L2 segment were as follows: flexion 6.36°; extension 8.12°; left bending 9.9°; right bending 7.85°; left rotation 4.61°; and right rotation 3.78°. The ROM results were comparable with those reported by (Alizadeh et al., 2013; Disch et al., 2007; Schmoelz et al., 2010) (Figure 3). To further verify the intact modle, we extracted the ROM of T11-L3 at 5.5N*M and compared it with the titro experiment. The ROM results were comparable with those reported by Couvertier et al., 2017 (Figure 3).

### ROM of the T12-L2 in nine FE models

The ROM values, including flexion, extension, left and right lateral bending, and left and right axial rotation, for the three internal fixation models in the upper, middle, and lower 1/3 of L1 fractures under different motion states were presented in Table 3. It was found that the P1-D1 fixation model showed maximum ROM in all six motion states, while the P2-D1 and P1-D2 fixation models showed no obvious difference in ROM values. It was also evident that the effect of the same fixation technique on ROM varied with different fracture locations. Across all models, flexion motion resulted in the highest ROM values with the maximum values of 11.63°, 12.85°, and 12.85° in the upper, middle, and lower 1/3 fractures, respectively, followed by extension, axial rotation, and the lowest ROM in left and right lateral bending. For the flexion motion, the P1D1, P2D1, and P1D2 fixation models could not provide sufficient stability for the fixed segments, resulting in significantly higher ROM values for all models compared to other physiological motion states.

**TABLE 3:** Range of motion (ROM) of the finite element models of different fixation strategies (°).

Fracture location	Upper 1/3 fracture			Middle 1/3 fracture			Lower 1/3 fracture		
Model/Motion	P1D1	P2D1	P1D2	P1D1	P2D1	P1D2	P1D1	P2D1	P1D2
Flexion	11.63	9.93	10.07	12.85	9.17	9.59	12.85	10.83	11.00
Extension	4.77	4.60	4.26	4.75	4.57	4.40	4.75	4.57	4.40
Left bending	1.35	0.74	0.63	0.82	0.70	0.65	1.72	0.70	0.63
Right bending	1.32	0.53	0.62	1.87	0.70	0.64	1.00	0.68	0.64
Left rotation	2.20	1.70	1.85	1.90	1.80	1.90	2.17	1.79	1.92
Right rotation	2.20	1.86	1.80	1.79	1.80	1.91	2.19	1.79	1.93

### Maximum von mises stress on the screws and rods

**FIGURE 4:**
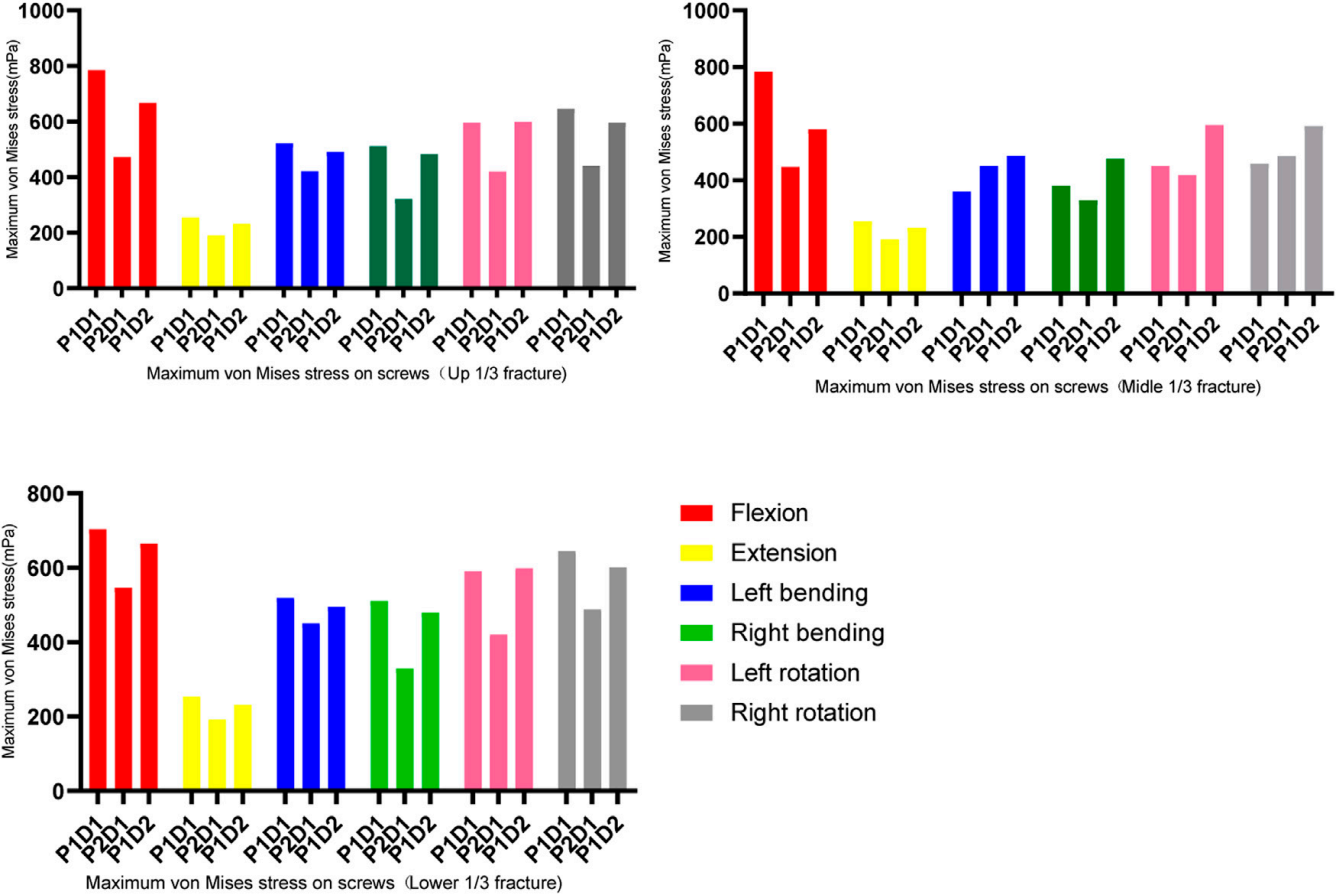
Maximum von Mises stress on the screws.

**FIGURE 5:**
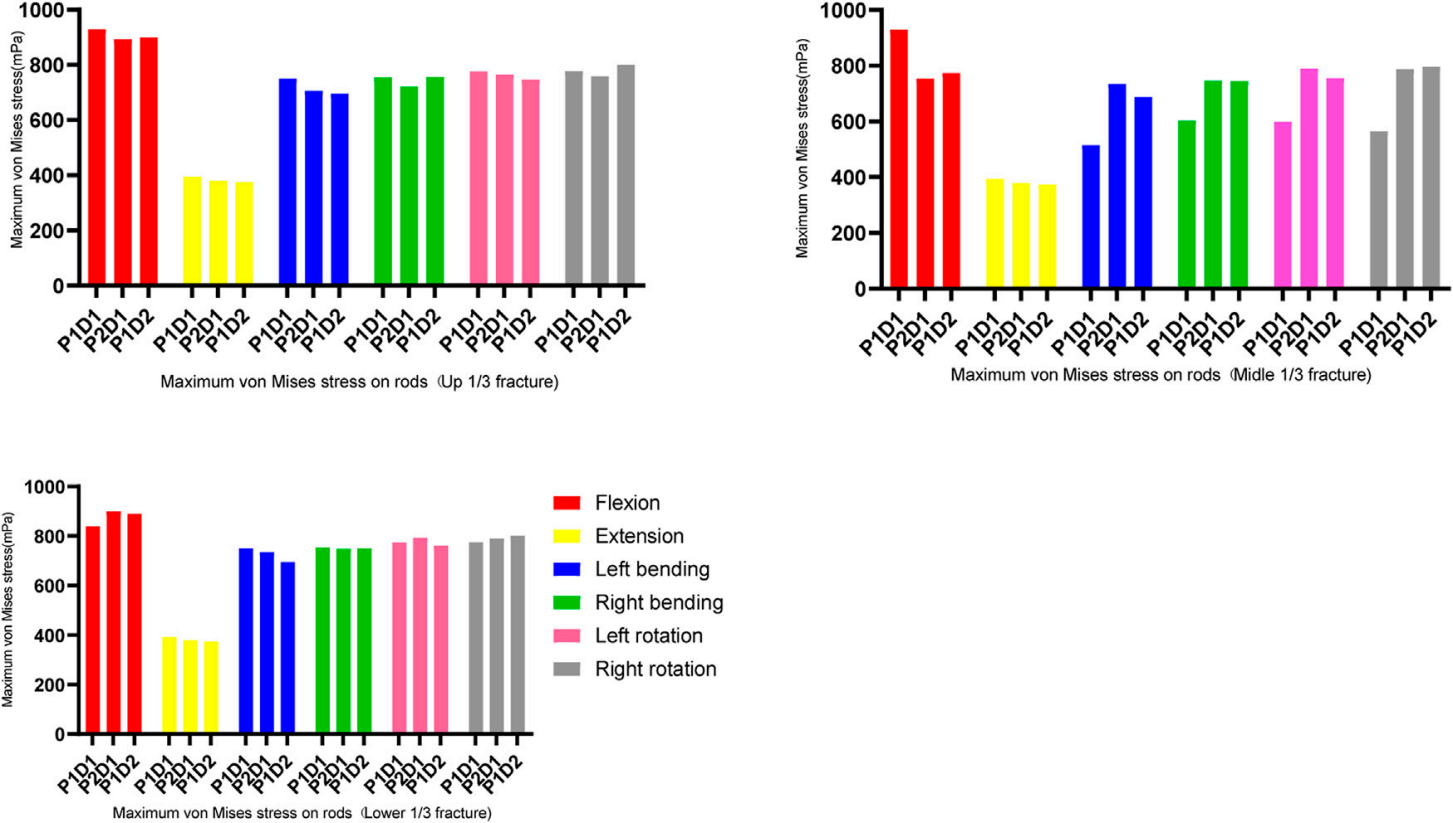
Maximum von Mises stress on the rods.

In all fixation models and states of motion, the maximum von Mises stress for both pedicle screws and rods was observed during flexion, while the lowest was during extension (as shown in Figures 4, 5). For upper and lower 1/3 fractures, the maximum von Mises stresses of pedicle screws in all three models were ranked in descending order as P1-D1, P1-D2 and P2-D1 models under all six physiological motion states, the maximum von Mises stresses in rods among the three models did not show significant differences. For middle 1/3 fractures, the maximum von Mises stresses in flexion and extension were identified in descending order as P1-D1, P1-D2 and P2-D1 models, however, the maximum von Mises stress values for pedicle screws were observed in the P1-D2 model, with slight differences between the P1-D1 and P2-D1 models, in lateral bending and axial rotation. Similarly, in flexion and extension, the maximum von Mises stress values for rods, were observed in the P1-D1 model, while in lateral bending and axial rotation, the von Mises stress value of the rod in the P1-D1 model was the smallest, and the maximum von Mises stress values of the rod in the P1-D2 and P2-D1 models did not show significant differences.

**FIGURE 6:**
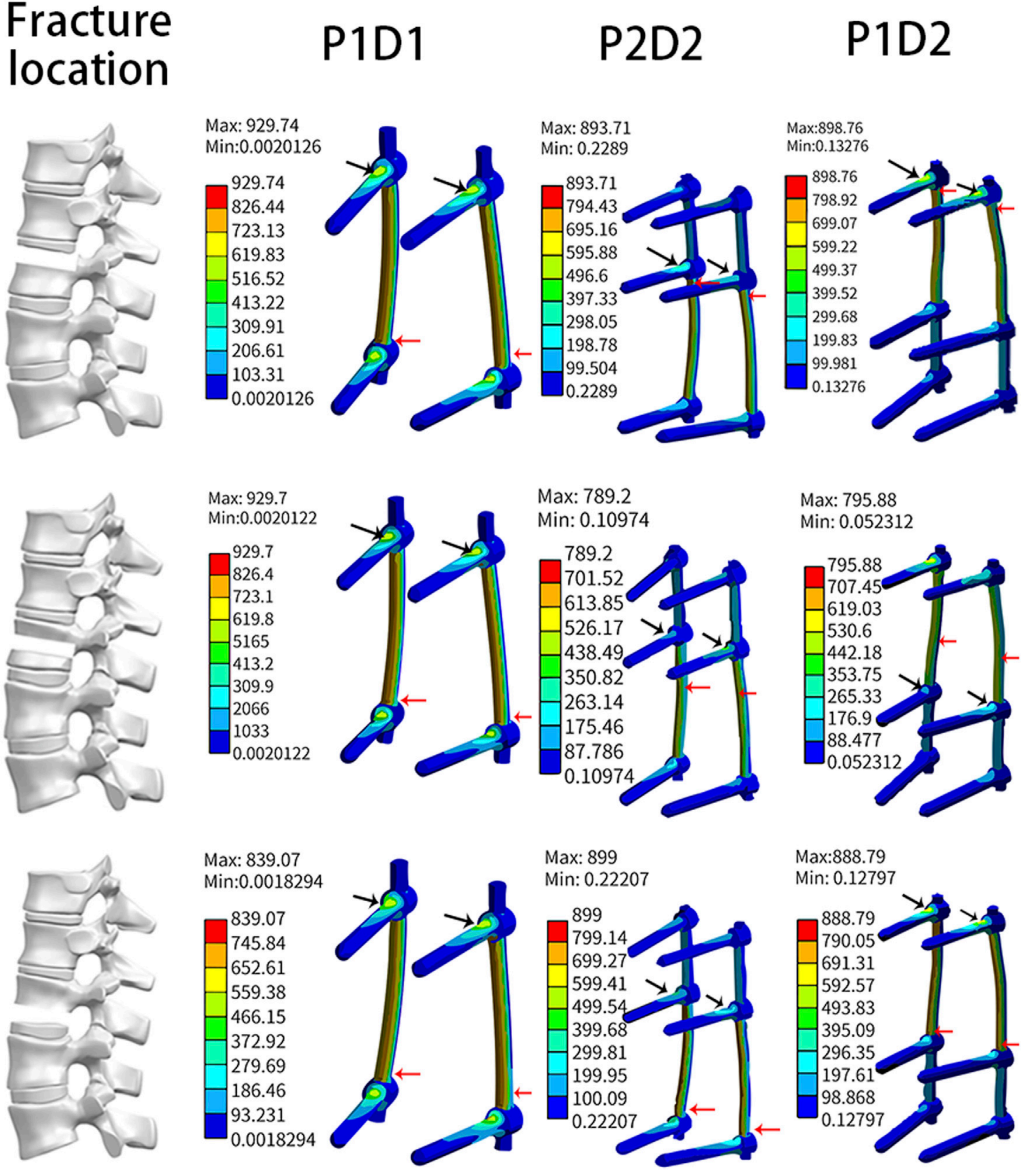
Maximum stress location on the screw and rod.

The results shown in Figure 6, indicate that the maximum von Mises stresses for pedicle screws of all three models were majorly concentrated around the screw roots, and the maximum stresses on the rods were located in the L1 cone region, i.e., at the fracture location.

## Discussion

To investigate the influence of sagittal location of vertebral body fracture on internal fixation system stress and the selection of surgical internal fixation strategy, 9 finite element models with three types of sagittal and three surgical strategies were developed in this study. The results showed in all the three sagittal models, P2D1 has a smaller ROM and less internal fixation stress. Therefore, the findings indicated that the sagittal location of the fracture does not affect the choice of surgical strategy, but can affect the level of mechanical stress on the internal fixation and their potential risk of failure.

Spinal fractures often occur in the thoracolumbar segment, and L1 is the most frequently involved site, taking up 16.2%–34.4% of all spinal fractures (Holmes et al., 2001; Katsuura et al., 2016; Leucht et al., 2009). Thus, we selected L1 to establish the FE model. There are various surgical strategies available, in addition to the P1D1, P2D1, and P1D2 methods mentioned in the article. Other options include the P2D2 and the combination of P1D1 with vertebral pedicle screw fixation, etc. However, if the pedicle of the injured vertebra is fractured, the screw could not be placed. Furthermore, it is difficult to establish the FE model because the screws are exposed in upper 1/3 fractures. It is thought that P2D2 does not significantly reduce stress on screws compared with P2D1 and P1D2. P2D1 involves the fixation of more segments, thereby resulting in loss of movement. P2D2 is not recommended as the first choice for the treatment of thoracolumbar fractures (Basaran et al., 2019; Wong et al., 2021). Therefore, in this study, we selected the P1D1, P2D1, and P1D2 fixation techniques.

Multiple posterior internal fixation surgical techniques have been widely used in clinical practice, with significant effects in the treatment of thoracolumbar fractures. However, controversy remains regarding the selection of surgical strategies (Cahueque et al., 2016). In recent years, studies have analyzed the biomechanical stability of fracture regions using different internal fixation models. The majority of these studies modeled the lower 1/3 of the vertebral body resection. Nevertheless, fractures can occur in the upper, middle, and lower regions (Denis, 1983; Rosenthal et al., 2018). Additionally, L1 is the most common site of thoracolumbar fractures. Therefore, T11-L3 was chosen to establish a fracture model of upper, middle, and lower 1/3 fractures of L1. Our results show that the sagittal distribution of fractures influences the ROM.

In all models, the largest ROM was obtained at the flexion motion. This finding is consistent with those noted in previous studies (Basaran et al., 2019). Under the flexion motion, fractures in the middle and upper 1/3 are associated with the shortest and longest ROM, respectively. For example, in the P1D1 fixation model, the ROM values for the upper, middle, and lower 1/3 were 11.63°, 9.93°, and 10.07°, respectively. The probable cause is that fractures located in the middle 1/3 have more uniform internal fixation stress is more uniform and greater stability. The pedicle plays an important role in the stability of the spine. When the fracture is located in the upper 1/3, the pedicle is involved, thereby increasing the ROM.

The biomechanical stability of fixation models is related to the extent and location of fixation (Wang et al., 2018). This study showed no significant difference in the ROM of extension, lateral bending, and axial rotation between the P2D1 and P1D2 models at the three fracture levels. The ROM values for both models were lower than those recorded for the P1D1 model at all six states of motion. This implies that six-screw fixation in the fracture area could provide more spinal stability than short-segment fixation. Theoretically, the addition of the fixation segment provides additional fixation points for fracture reduction and kyphosis correction. This is consistent with the conclusions of previous studies (Jindal et al., 2020; Xu et al., 2019).

In all models, the maximum screw stress was obtained under flexion motion. The distance between the fracture position and the screw affects the instrument stress (Zhang et al., 2021). Our findings showed that, under the same type of internal fixation, the fracture location altered (reduced or increased) the stress on the screw but did not affect the maximum stress location (Figures 4, 6).

It has been documented that stress concentration occurs in adjacent segments under short-segment fixation. This may cause looseness and breakage due to fatigue by increased bearing stress of the internal fixation (Jindal et al., 2020). The results of this study further indicated that an increase in the number of screws can reduce the average stress, thus reducing the risk of screw breakage (Xu et al., 2019). In the three fracture distributions analyzed in this study, the maximum stress of P1D2 and P2D1 was significantly lower than that of P1D1. This result was comparable with those reported in previous studies (Wu et al., 2019). Our results showed that P2D1 was considered more appropriate in this setting than P1D2, comparable with previous studies (C. E. Wong et al., 2021). Clinically, this method has proven to be an effective alternative for fixation (Modi et al., 2009). However, they did not consider the sagittal location variation of the fractures.

The fracture site was the most unstable region of the constructs. Hence the maximum stress on the rods occurred across the fractures site that was in line with previous studies (Wong et al., 2021). In the present study, the site of maximum stress on the rod shifted downward in parallel with the location of the fracture, and the stress value changed accordingly. For the three fixation strategies, the maximum stress on the instrument in the upper and lower 1/3 fracture models was similar. In the middle 1/3 fracture model, P2D1 and P1D1 were associated with similar stress. The stress noted for these techniques was higher than that recorded for P1D1. These findings could guide physicians regarding the clinical management of type A3 fractures.

This study had several limitations. Although finite element analysis is a valid method in biomechanical studies, it still does not fully simulate the comparison of human treatments. Experimental results of finite element analysis represent a new clinical trend rather than definitive conclusions (Lewis et al., 2021). Under 7.5 N*M moments, the fracture models may seriously deform. We did not validate the fracture models. In this study pertaining to the somewhat simplified and idealized material properties used in the simulation, such as the nonlinear behavior of spinal ligaments, the viscoelasticity of intervertebral discs, and the varying degrees of degeneration - all of which differ from cadaveric specimens. And what’s more, pure structural resection does not reflect the complexity of fracture morphology. Only the A3 fracture models were established in this study and the boundary conditions in terms of the complex segmental motion of the human spine in the thoracolumbar segment were simplified Thus, models with actual ROM and with other fracture subtypes should be warranted in the future. The screw thread size should be considered for more realistic screw stress analyses in future studies. In addition, using a nonlinear material constitutive model is necessary to study the localized failure of internal fixation systems. However, the stress trends for the different procedures observed in this study are comparable to previous studies. Finally, although there are numerous posterior internal fixation methods used to treat fractures, only three techniques were modeled in this study. Further investigations should be performed to evaluate more biomechanical properties of other models concerning other posterior internal fixation methods.

## Conclusion

The sagittal location of fractures did not affect the choice of surgical strategies; however, it affected the magnitude of stress and distribution of the internal fixation system.

## Data Availability

The raw data supporting the conclusion of this article will be made available by the authors, without undue reservation.

## References

[B1] Alizadeh M. Kadir M. R. A. Fadhli M. M. Fallahiarezoodar A. Azmi B. Murali M. R. (2013). The use of X-shaped cross-link in posterior spinal constructs improves stability in thoracolumbar burst fracture: A finite element analysis. J. Orthop. Res. 31 (9), 1447–1454. 10.1002/jor.22376 23640802

[B2] Basaran R. Efendioglu M. Kaksi M. Celik T. Mutlu I. Ucar M. (2019). Finite element analysis of short-versus Long-Segment posterior fixation for thoracolumbar burst fracture. World Neurosurg. 128, e1109–e1117. 10.1016/j.wneu.2019.05.077 31103754

[B3] Cahueque M. Cobar A. Zuniga C. Caldera G. (2016). Management of burst fractures in the thoracolumbar spine. J. Orthop. 13 (4), 278–281. 10.1016/j.jor.2016.06.007 27408503 PMC4930335

[B4] Couvertier M. Germaneau A. Saget M. Dupré J. Doumalin P. Brémand F. (2017). Biomechanical analysis of the thoracolumbar spine under physiological loadings: Experimental motion data corridors for validation of finite element models. Proc. Institution Mech. Eng. Part H J. Eng. Med. 231 (10), 975–981. 10.1177/0954411917719740 28707505

[B5] den Ouden L. P. Smits A. J. Stadhouder A. Feller R. Deunk J. Bloemers F. W. (2019). Epidemiology of spinal fractures in a level one trauma center in The Netherlands. Spine 44 (10), 732–739. 10.1097/BRS.0000000000002923 30395086

[B6] Denis F. (1983). The three column spine and its significance in the classification of acute thoracolumbar spinal injuries. Spine (Phila Pa 1976) 8 (8), 817–831. 10.1097/00007632-198311000-00003 6670016

[B7] Disch A. C. Luzzati A. Melcher I. Schaser K. D. Feraboli F. Schmoelz W. (2007). Three-dimensional stiffness in a thoracolumbar en-bloc spondylectomy model: A biomechanical *in vitro* study. Clin. Biomech. 22 (9), 957–964. 10.1016/j.clinbiomech.2007.07.010 17854958

[B8] El B. H. Saleh A. K. Elsherief F. Abuomira I. Elkawary A. I. (2020). Short-Segment fixation of thoracolumbar fractures with incorporated screws at the level of fracture. Orthop. Surg. 12 (1), 170–176. 10.1111/os.12590 31916389 PMC7031547

[B9] Galbusera F. Qian Z. Casaroli G. Bassani T. Costa F. Schlager B. (2018). The role of the size and location of the tumors and of the vertebral anatomy in determining the structural stability of the metastatically involved spine: A finite element study. Transl. Oncol. 11 (3), 639–646. 10.1016/j.tranon.2018.03.002 29604509 PMC6054594

[B10] Girardo M. Masse A. Risitano S. Fusini F. (2021). Long versus short segment instrumentation in osteoporotic thoracolumbar vertebral fracture. Asian Spine J. 15 (4), 424–430. 10.31616/asj.2020.0033 33059438 PMC8377206

[B11] Guo H. Li J. Gao Y. Nie S. Quan C. Li J. (2021). A finite element study on the treatment of thoracolumbar fracture with a new spinal fixation system. Biomed. Res. Int. 2021, 1–9. 10.1155/2021/8872514 33937413 PMC8055395

[B12] Guo L. X. Li W. J. (2019). A biomechanical investigation of thoracolumbar burst fracture under vertical impact loads using finite element method. Clin. Biomech. (Bristol, Avon) 68, 29–36. 10.1016/j.clinbiomech.2019.05.018 31146081

[B13] Holmes J. F. Miller P. Q. Panacek E. A. Lin S. Horne N. S. Mower W. R. (2001). Epidemiology of thoracolumbar spine injury in blunt trauma. Acad. Emerg. Med. 8 (9), 866–872. 10.1111/j.1553-2712.2001.tb01146.x 11535478

[B14] Jindal R. Jasani V. Sandal D. Garg S. K. (2020). Current status of short segment fixation in thoracolumbar spine injuries. J. Clin. Orthop. Trauma 11 (5), 770–777. 10.1016/j.jcot.2020.06.008 32879564 PMC7452221

[B15] Katsuura Y. Osborn J. M. Cason G. W. (2016). The epidemiology of thoracolumbar trauma: A meta-analysis. J. Orthop. 13 (4), 383–388. 10.1016/j.jor.2016.06.019 27504058 PMC4963326

[B16] Leucht P. Fischer K. Muhr G. Mueller E. J. (2009). Epidemiology of traumatic spine fractures. Injury 40 (2), 166–172. 10.1016/j.injury.2008.06.040 19233356

[B17] Lewis G. S. Mischler D. Wee H. Reid J. S. Varga P. (2021). Finite element analysis of fracture fixation. Curr. Osteoporos. Rep. 19, 403–416. 10.1007/s11914-021-00690-y 34185266 PMC8422380

[B18] Liang C. Liu B. Zhang W. Yu H. Cao J. Yin W. (2020). Clinical effects of posterior limited Long-Segment pedicle instrumentation for the treatment of thoracolumbar fractures. J. Invest. Surg. 33 (1), 25–30. 10.1080/08941939.2018.1474301 29856666

[B19] Liu B. Zhu Y. Liu S. Chen W. Zhang F. Zhang Y. (2018). National incidence of traumatic spinal fractures in China. Medicine 97 (35), e12190. 10.1097/MD.0000000000012190 30170470 PMC6393073

[B20] Modi H. N. Chung K. J. Seo I. W. Yoon H. S. Hwang J. H. Kim H. K. (2009). Two levels above and one level below pedicle screw fixation for the treatment of unstable thoracolumbar fracture with partial or intact neurology. J. Orthop. Surg. Res. 4, 28. 10.1186/1749-799X-4-28 19635134 PMC2724433

[B21] Mu S. Wang J. Gong S. (2022). Mechanical analysis of posterior pedicle screw system placement and internal fixation in the treatment of lumbar fractures. Comput. Math. Methods Med. 2022, 1–10. 10.1155/2022/6497754 PMC9017477 35450206

[B22] Naoum S. Vasiliadis A. V. Koutserimpas C. Mylonakis N. Kotsapas M. Katakalos K. (2021). Finite element method for the evaluation of the human spine: A literature overview. J. Funct. Biomater. 12, 43. 10.3390/jfb12030043 34449646 PMC8395922

[B23] Park W. M. Kim K. Kim Y. H. (2013). Effects of degenerated intervertebral discs on intersegmental rotations, intradiscal pressures, and facet joint forces of the whole lumbar spine. Comput. Biol. Med. 43 (9), 1234–1240. 10.1016/j.compbiomed.2013.06.011 23930818

[B24] Petersilge C. A. Emery S. E. (1996). Thoracolumbar burst fracture: Evaluating stability. Semin. Ultrasound CT MR 17 (2), 105–113. 10.1016/s0887-2171(96)90010-4 8845195

[B25] Rohlmann A. Zander T. Rao M. Bergmann G. (2009). Realistic loading conditions for upper body bending. J. Biomech. 42 (7), 884–890. 10.1016/j.jbiomech.2009.01.017 19268291

[B26] Rosenthal B. D. Boody B. S. Jenkins T. J. Hsu W. K. Patel A. A. Savage J. W. (2018). Thoracolumbar burst fractures. Clin. Spine Surg. 31 (4), 143–151. 10.1097/BSD.0000000000000634 29578877

[B27] Schmoelz W. Schaser K. D. Knop C. Blauth M. Disch A. C. (2010). Extent of corpectomy determines primary stability following isolated anterior reconstruction in a thoracolumbar fracture model. Clin. Biomech. 25 (1), 16–20. 10.1016/j.clinbiomech.2009.09.010 19837494

[B28] Vaccaro A. R. Oner C. Kepler C. K. Dvorak M. Schnake K. Bellabarba C. (2013). AOSpine thoracolumbar spine injury classification system: Fracture description, neurological status, and key modifiers. Spine (Phila Pa 1976) 38 (23), 2028–2037. 10.1097/BRS.0b013e3182a8a381 23970107

[B29] Wang H. Mo Z. Han J. Liu J. Li C. Zhou Y. (2018). Extent and location of fixation affects the biomechanical stability of short- or long-segment pedicle screw technique with screwing of fractured vertebra for the treatment of thoracolumbar burst fractures: An observational study using finite element analysis. Med. Baltim. 97 (26), e11244. 10.1097/MD.0000000000011244 PMC6039687 29952989

[B30] Wang P. Hu X. (2020). Biomechanical finite element analysis of superior endplate collapse after thoracolumbar fracture surgery. Ann. Transl. Med. 8 (12), 753. 10.21037/atm-20-4091 32647678 PMC7333103

[B31] Wang S. Park W. M. Gadikota H. R. Miao J. Kim Y. H. Wood K. B. (2013). A combined numerical and experimental technique for estimation of the forces and moments in the lumbar intervertebral disc. Comput. Method. Biomec. 16 (12), 1278–1286. 10.1080/10255842.2012.668537 PMC3429631 22551235

[B32] Wong C. E. Hu H. T. Tsai C. H. Li J. L. Hsieh C. C. Huang K. Y. (2021). Comparison of posterior fixation strategies for thoracolumbar burst fracture: A finite element study. J. Biomech. Eng. 143 (7), 071007. 10.1115/1.4050537 33729440

[B33] Wong C. Gehrchen P. M. Darvann T. Kiaer T. (2003). Nonlinear finite-element analysis and biomechanical evaluation of the lumbar spine. IEEE Trans. Med. Imaging 22 (6), 742–746. 10.1109/TMI.2003.814783 12872949

[B34] Wu Y. Chen C. H. Tsuang F. Y. Lin Y. C. Chiang C. J. Kuo Y. J. (2019). The stability of long-segment and short-segment fixation for treating severe burst fractures at the thoracolumbar junction in osteoporotic bone: A finite element analysis. PLoS One 14 (2), e0211676. 10.1371/journal.pone.0211676 30716122 PMC6361511

[B35] Xu M. Yang J. Lieberman I. Haddas R. (2019). Stress distribution in vertebral bone and pedicle screw and screw-bone load transfers among various fixation methods for lumbar spine surgical alignment: A finite element study. Med. Eng. Phys. 63, 26–32. 10.1016/j.medengphy.2018.10.003 30344069

[B36] Zhang T. Wang Y. Zhang P. Xue F. Zhang D. Jiang B. (2021). Different fixation pattern for thoracolumbar fracture of ankylosing spondylitis: A finite element analysis. PLoS One 16 (4), e0250009. 10.1371/journal.pone.0250009 33836027 PMC8034711

